# Predicting Disease Risk Using Bootstrap Ranking and Classification Algorithms

**DOI:** 10.1371/journal.pcbi.1003200

**Published:** 2013-08-22

**Authors:** Ohad Manor, Eran Segal

**Affiliations:** 1 Dept. of Computer Science and Applied Mathematics, Weizmann Institute of Science, Rehovot, Israel; 2 Department of Molecular Cell Biology, Weizmann Institute of Science, Rehovot, Israel; Memorial Sloan-Kettering Cancer Center, United States of America

## Abstract

Genome-wide association studies (GWAS) are widely used to search for genetic loci that underlie human disease. Another goal is to predict disease risk for different individuals given their genetic sequence. Such predictions could either be used as a “black box” in order to promote changes in life-style and screening for early diagnosis, or as a model that can be studied to better understand the mechanism of the disease. Current methods for risk prediction typically rank single nucleotide polymorphisms (SNPs) by the p-value of their association with the disease, and use the top-associated SNPs as input to a classification algorithm. However, the predictive power of such methods is relatively poor. To improve the predictive power, we devised *BootRank*, which uses bootstrapping in order to obtain a robust prioritization of SNPs for use in predictive models. We show that BootRank improves the ability to predict disease risk of unseen individuals in the Wellcome Trust Case Control Consortium (WTCCC) data and results in a more robust set of SNPs and a larger number of enriched pathways being associated with the different diseases. Finally, we show that combining BootRank with seven different classification algorithms improves performance compared to previous studies that used the WTCCC data. Notably, diseases for which BootRank results in the largest improvements were recently shown to have more heritability than previously thought, likely due to contributions from variants with low minimum allele frequency (MAF), suggesting that BootRank can be beneficial in cases where SNPs affecting the disease are poorly tagged or have low MAF. Overall, our results show that improving disease risk prediction from genotypic information may be a tangible goal, with potential implications for personalized disease screening and treatment.

## Author Summary

Genome-wide association studies are widely used to search for genetic loci that underlie human disease. Another goal is to predict disease risk for different individuals given their genetic sequence. Such predictions could either be used as a “black box” in order to promote changes in life-style and screening for early diagnosis, or as a model that can be studied to better understand the mechanism of the disease. Current methods for risk prediction have relatively poor performance, with one possible explanation being the fact they rely on a noisy ranking of genetic variants given to them as input. To improve the predictive power, we devised *BootRank*, a ranking method less sensitive to noise. We show that BootRank improves the ability to predict disease risk of unseen individuals in the Wellcome Trust Case Control Consortium (WTCCC) data, and that combining BootRank with different classification algorithms improves performance compared to previous studies that used these data. Overall, our results show that improving disease risk prediction from genotypic information may be a tangible goal, with potential implications for personalized disease screening and treatment.

## Introduction

Genome-wide association studies (GWAS) have recently become the dominant method for searching for the genetic basis that underlies human diseases [1]–[3]. A typical GWAS consists of a collection of genotypes from affected (cases) and healthy (controls) individuals, allowing researches to search for single nucleotide polymorphisms (SNPs) that significantly differ in frequencies between the two groups [4], [5]. Such studies have identified over 1,000 loci associated with more than 165 diseases and traits [6], [7], such as diabetes [8]–[10], cancer [11]–[13], and rheumatoid arthritis [14].

In an effort to move beyond single SNP associations, multiple studies tried to predict disease risk in individuals based on their SNP profiles [15]–[19]. In such risk prediction studies, the entire set of SNPs is potentially used to estimate the risk of every individual to suffer from the disease, and this risk is then compared with the actual disease status of the individual (e.g., case/control). The quality of the prediction is assessed in several ways, with the AUC value (area under the receiver operating curve) being a popular choice, albeit not a perfect one [18], [20]. Intuitively, the AUC can be thought of as the probability that a predictor will correctly classify a pair of samples, one positive and one negative, with a perfect predictor having an AUC of 1, and a random predictor having an AUC of 0.5. Currently, GWAS-based predictors achieve a broad range of AUCs, ranging from relatively high AUC values such as ∼0.9 for Type 1 diabetes, and near random AUC values for other diseases such as mood disorders [18].

Here, we set out to improve our ability to predict disease risk of individuals based only on their SNP genotypes. In risk prediction algorithms, a large set of SNPs is used to perform the predictions, with the identity of the selected SNPs varying due to noise in the choice of data. We thus hypothesized that improvements to risk prediction may be achieved by selecting SNPs that are less sensitive to noise and to the exact choice of data. To test this idea, we devised *BootRank*, a method that uses bootstrapping in order to rank SNPs for use within predictive models. In BootRank, the data is re-sampled multiple times and a SNP ranking is produced for each such sample, with the final SNP ranking being an aggregate of all the sample rankings.

We tested BootRank on the Wellcome Trust Case Control Consortium (WTCCC) data [21] and found that it increases the robustness of the top-ranked SNPs across different cross-validation sets. In order to validate that the SNP ranking produced by BootRank is also more biologically relevant, we compared its ranking to that based on GWAS association p-values (termed *GWASRank*). We found that BootRank results in a larger number of enriched pathways associated with the different diseases, and that the pathways detected have substantial support in the literature. Finally, we used the SNP rankings of either BootRank or GWASRank as inputs to seven different classification algorithms and found that using BootRank significantly improves the predictive power for held-out test individuals. Notably, the diseases where using BootRank improves performance the most were recently found to have an underestimated value of heritability, likely because they are predominately affected by variants that have low minimum allele frequency (MAF) [22]. This unexpected finding suggests that BootRank is especially beneficial in cases were the underlying SNPs that affect the disease are poorly tagged or have low MAF.

In summary, our results highlight the importance of robust SNP ranking in the task of disease risk prediction, and offer a concrete method to improve ranking robustness, and consequently the power to predict disease risk and identify biological pathways that may play a role in the different diseases.

## Results

### Bootstrap ranking increases the fraction of top SNPs that overlap between different cross-validations

Since current disease risk predictors are highly dependent on the initial SNP ranking they take as input, we hypothesized that a common limitation in these predictors may be the sensitivity of the ranking to the exact choice of data. We thus wished to test whether we can improve the ability to predict risk by selecting SNPs that are less sensitive to noise and the exact choice of data. To achieve such robust ranking of SNPs, we used bootstrapping [23], which uses resampling of the data in order to overcome noise. Bootstrapping makes the assumption that individuals are independent and identically distributed, which could be problematic for scenarios such as familial datasets. In the WTCCC dataset, however, individuals have no known dependencies [21]. In our method, we resample the data multiple times, producing a SNP ranking for each such sampling based on GWAS p-values, and then aggregate rankings from all samples to produce a final SNP ranking. We compared our bootstrapping SNP ranking method (termed *BootRank*), with the commonly used GWAS p-value SNP ranking (termed *GWASRank*) in a strict cross-validation analysis.

When using cross-validation (CV) to generate training and test sets, there are multiple training sets formed in the process (e.g., in a 5-fold CV, there are 5 different training sets, with a 75% overlap of individuals between every pair of training sets). The fraction of top SNPs that overlap between different training sets when ranking is performed either by *GWASRank* (as commonly done), or by *BootRank*, is indicative of the robustness of the SNP ranking method.

To test the robustness of both ranking methods, we used the Wellcome Trust Case Control Consortium (WTCCC) data consisting of ∼2000 cases and ∼1500 control genotypes for 7 different diseases (T1D, Type 1 diabetes; T2D, Type 2 diabetes; CD, Crohn's disease; CAD, coronary artery disease; BD, bipolar disorder; RA, rheumatoid arthritis; HT, hypertension). To ensure minimal bias in the genotypes, we removed all SNPs that were excluded in the original WTCCC paper [21], [24], including SNPs with deviation from Hardy-Weinberg equilibrium or bad clustering (see methods). For each disease, we randomly split the data into training and test sets using a 5-fold CV partition scheme. Next, in each training set, we computed for each SNP the minimum (best) p-value it obtains in one of the genetic association tests (i.e., general, dominant, recessive and additive, see **Methods**) and ranked SNPs accordingly, as usually done in GWAS studies (i.e., by *GWASRank*). In addition, we employed a bootstrapping approach, where we re-sample our data from the training set and produce a p-value based ranking for each such sample, and aggregate all rankings to a final SNP ranking based on the median rank SNPs obtained in all bootstrap samples (i.e., by *BootRank*). To ensure that our results are not due to a specific random partition, we repeated this 5-fold CV analysis 10 times and reported the overall averages.

**Figure 1:**
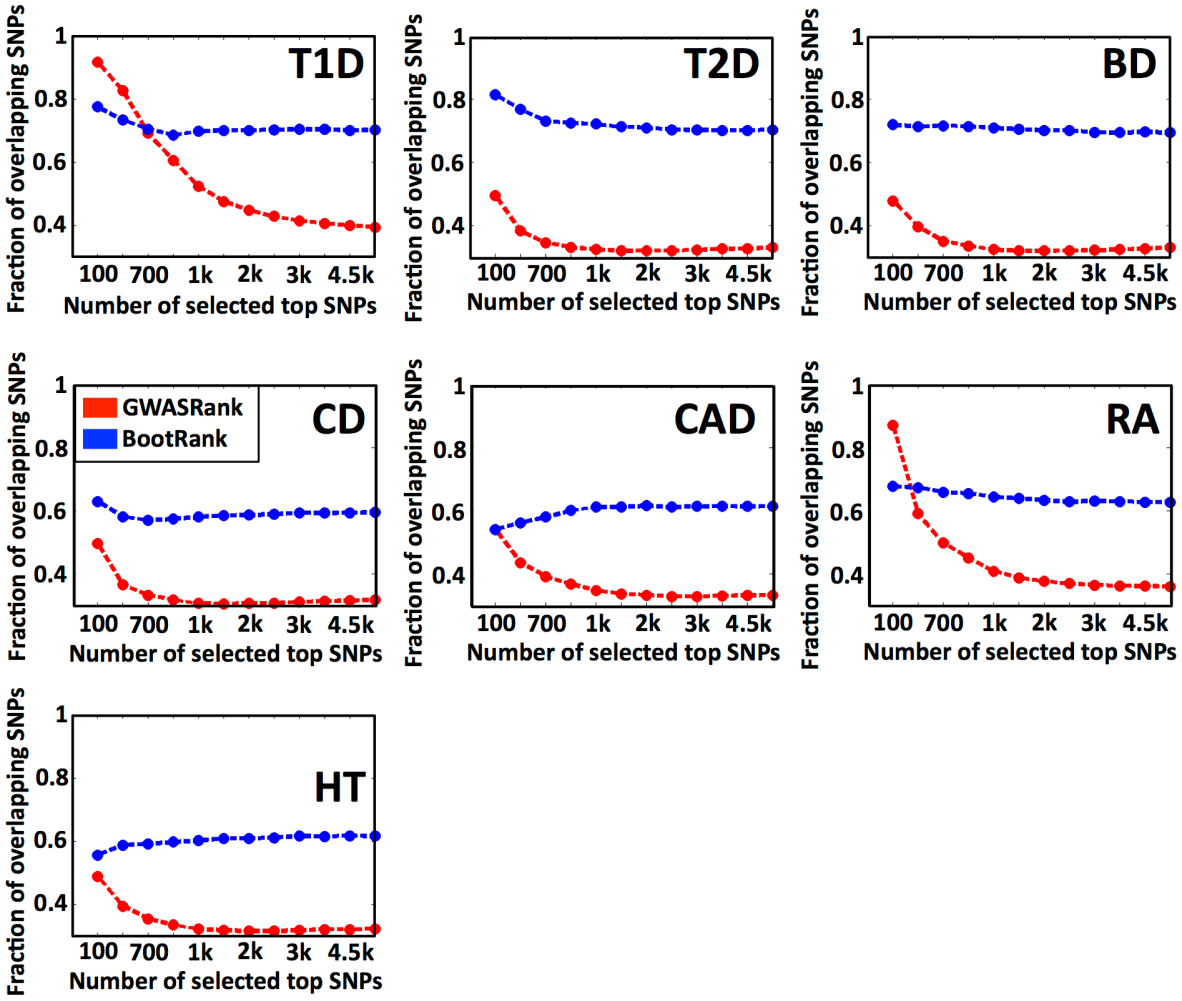
Fraction of intersection of filtered SNPs lists between different cross-validation partitions. For each disease (T1D, Type 1 diabetes; T2D, Type 2 diabetes; CD, Crohn's disease; CAD, coronary artery disease; BD, bipolar disorder; RA, rheumatoid arthritis; HT, hypertension), shown is the mean fraction (y-axis) of top SNPs shared between training sets from different cross-validations when ranking SNPs by GWASRank (red) or BootRank (blue). The x-axis shows the number of SNPs that were selected as top SNPs from the SNP ranking.

We found that across all 7 diseases examined, a significantly higher fraction of the top SNPs are shared between different CV training sets when using BootRank as compared to GWASRank (**Figure 1**). In addition, the fraction of overlapping SNPs for GWASRank decreases as more SNPs are employed (e.g., from 50% for 100 SNPs to 30% for 2000 SNPs in Bipolar disorder (BD)), whereas the fraction of overlapping SNPs in BootRank remains relatively constant when the number of SNPs increases (e.g., ∼70% in BD regardless of the number of input SNPs). We also found that the advantage of BootRank becomes larger if a smaller sample size is used to rank the SNPs (e.g., if only 25% of data is used to rank SNPs for T2D, GWASRank shows ∼0% overlap while BootRank shows ∼75% overlap, **Figure S8**), further attesting to the robustness of BootRank.

These results show that the fraction of top SNPs that overlap between different training sets using GWASRank is rather low, and that using BootRank is beneficial for increasing the robustness of SNP ranking across multiple CVs in 7 different diseases. However, since ranking robustness by itself is meaningless (e.g., as in ranking SNPs lexicographically by their ID), we next sought to test whether this more robust ranking also has merit in the biological sense.

### Bootstrap ranking increases the number of enriched pathways detected

To independently validate the biological relevance of our SNP ranking, we searched for enriched biological pathways in the different diseases. A popular method to detect pathways that are important to a specific disease is to rank genes according to their expected association, and then use this ranking to compute an enrichment p-value for different pathways (e.g., KEGG pathways). The gene rankings tend to be based on the p-values obtained by GWAS, either by assigning to each gene the best p-value obtained by one of its nearby SNPs [25]–[28], or by pooling all p-values of SNPs inside a gene to one combined p-value [29], [30]. However, in both methods, the initial GWAS p-values are critical, and we thus tested whether the more robust ranking of BootRank also improves the enrichment of pathways in the different diseases.

**Figure 2:**
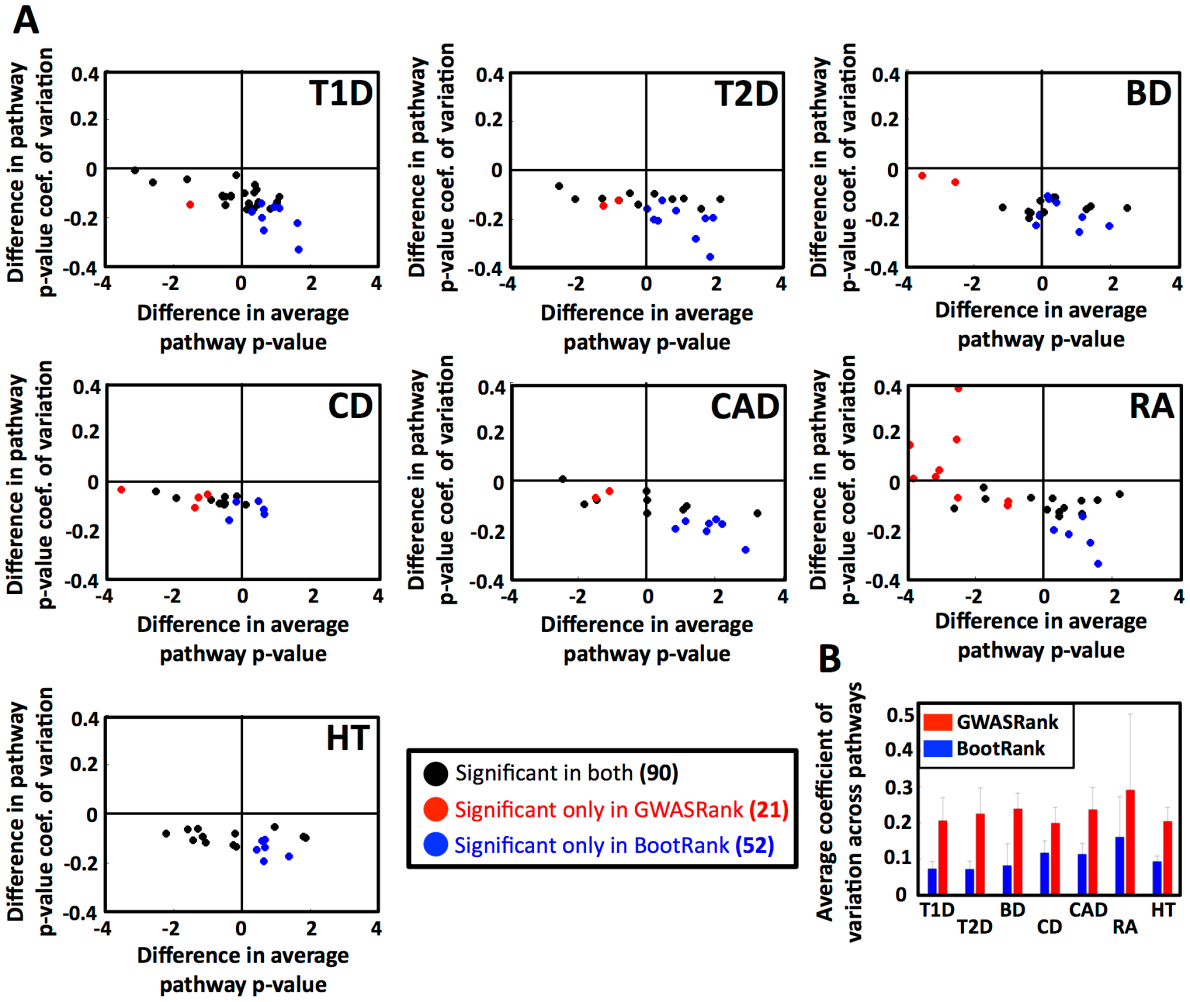
BootRank reduces noise in p-value pathway enrichment scores and detects more enriched pathways. (a) For each disease (T1D, Type 1 diabetes; T2D, Type 2 diabetes; CD, Crohn's disease; CAD, coronary artery disease; BD, bipolar disorder; RA, rheumatoid arthritis; HT, hypertension), shown are the differences in the average (x-axis) and coefficient of variation (y-axis) of the enrichment p-values of all significantly enriched KEGG pathways. (b) The mean noise (measured as coefficient of variation) of pathway enrichment p-values are shown for all diseases for GWASRank (red) or BootRank (blue).

For each disease and CV training set, we first ranked all the SNPs using either GWASRank or BootRank. Next, we assigned to each gene the best p-value or bootstrap rank obtained by one of the SNPs that resides within it or within its flanking 5 kb, resulting in a ranked list of genes (a total of 172,854 SNPs were mapped to 13,294 genes). We then computed an enrichment p-value for each KEGG [31], [32] pathway using the Wilcoxon rank-sum test, and defined a pathway as enriched if it passed the p-value threshold of P<0.01 in at least 90% of all CV training sets (**Figure 2**). We found that ranking by BootRank reduced the noise of computed p-values across different CV training sets (as measured by the p-value's coefficient of variation) for 155/163 (95%) of enriched pathways. In addition, BootRank increased the overall number of enriched KEGG pathways in 6 of 7 diseases (**Figure 2**, a total of 52 uniquely enriched pathways in BootRank compared with 21 in GWASRank).

Next, we examined whether the enriched pathways are known to affect the corresponding disease. In Type 1 diabetes (T1D, **Table S1**), three interesting pathways that were enriched only in BootRank were “*Valine, leucine and isoleucine biosynthesis*”, “*Chemokine signaling pathway*”, and “*MAPK signaling pathway*”. Notably, a paper that studied longitudinal changes in the amino acid profile in T1D mice, found that the plasma concentrations of valine, leucine, and isoleucine were significantly higher in the diabetic mice [33]. In addition, a few works showed that people with high risk of developing T1D have abnormal level of chemokines [34], and that higher expression levels of the chemokine receptor accelerated disease progress in mice [35]. Another paper also found an enrichment of the MAPK pathway in T1D [36].

In Type 2 diabetes mellitus (T2DM, **Table S2**), pathways that were enriched only in BootRank included “*Type II diabetes mellitus*”, “*Caffeine metabolism*”, “*Complement and coagulation cascades*” and “*alpha-Linolenic acid metabolism*”. The “*Type II diabetes mellitus*” pathway is connected to the disease since it is in fact based on multiple studies of T2DM [37]. In addition, several works showed that caffeine intake affects glucose levels and T2DM risk [38]–[40]. Genes involved in coagulation were found to be upregulated in T2DM patients [41], [42], and consumption of alpha-Linolenic acids was found to ameliorate features of T2DM [43].

In Bipolar disorder (BD, **Table S3**) the “*Neuroactive ligand-receptor interaction*” and “*Propanoate metabolism*” pathways were enriched only in BootRank. The Neuroactive ligand-receptor interaction pathway was indeed found to be enriched in a GWAS replication study [44], and in a study listing potential targets for novel therapeutics for BD, one of the suggested targets was a glutamate propionic acid receptor [45], which is part of the Propanoate metabolism pathway.

In Coronary artery disease (CAD, **Table S4**) several pathways had enrichment only in BootRank including “*Type II diabetes mellitus*”, “*Colorectal cancer*” and “*Endometrial cancer*”. Notably, there is evidence that all three diseases are also associated with increased levels of vascular diseases [46]–[49].

In Hypertension (HT, **Table S5**), “*Amyotrophic lateral sclerosis (ALS)*” and “*Ether lipid metabolism*” pathways were enriched only in BootRank. A recent paper showed that ALS patients have higher frequency of HT [50], and another paper found an Ether lipid deficiency in the blood plasma of HT patients [51].

In Crohn's disease (CD, **Table S6**), pathways unique to BootRank, included “*Starch and sucrose metabolism*”, “*Pantothenate and CoA biosynthesis*”, “*MAPK signaling pathway*”, and “*Neuroactive ligand-receptor interaction*”. Several studies showed that patients with CD have higher intake of starch and sugar [52], and that a low-starch diet can be beneficial for them [53]. In addition, the mRNA and protein levels of Acyl-CoA-synthetase-5 were found to be substantially reduced in CD patients [54], and MAPKs were found to be critically involved in the pathogenesis of Crohn's disease [55]. Interestingly, a new drug intended to treat patients with irritable bowel syndrome targets the GABAA-receptor gene [56], which is part of the Neuroactive ligand-receptor pathway.

In rheumatoid arthritis (RA, **Table S7**), among many pathways that were enriched only in BootRank were also “*Melanogenesis*”, “*Wnt signaling pathway*” and “*Hedgehog signaling pathway*”. In a study of the regulation of melanin pigmentation, it was shown that patients with RA show localized increased levels of β-Endorphin, which in turn had been implicated in skin pathogenesis [57]. In addition, Wnt signaling and the hedgehog pathway were found to be implicated in RA in mice and humans [58], [59].

Thus, these pathway enrichments show that using BootRank can reduce the noise in p-value computations, allowing more enriched pathways to be detected. Moreover, the enriched pathways have considerable support in the literature as being involved in the various diseases. These results show that BootRank SNP ranking is not only more robust, but also more biologically relevant than that of GWASRank.

### Bootstrap ranking of SNPs results in better disease risk prediction

Next, we tested whether BootRank's more robust ranking of SNPs can also improve risk prediction of held-out test individuals. To this end, for each disease we used the SNP ranking based only on the training data to filter the top SNPs at some given threshold (e.g., top 1000 SNPs), and then used these SNPs to learn a predictive discriminative model on training individuals using seven different classification algorithms: (1) Random forest (RF) [60], an ensemble classifier that consists of many decision trees; (2) Regularized logistic regression (RLR) [61], where the solution is a sparse vector of weights over the features; (3) A support vector machine (SVM) [62] that uses weights on the training examples to classify test cases; (4) Naïve Bayes (NB), a probabilistic classifier based on applying Bayes' rule with independence assumptions; (5) Robust adaboost (RAB), an adaptive ensemble algorithm where new classifiers are tweaked in favor of those instances misclassified by previous classifiers; (6) Allele count (AC), where classification is done based on counting risk alleles in each individual; and (7) Log odds (LO), where the classification is also based on the frequencies of alleles in cases and controls.

We ranked SNPs either by BootRank or by GWASRank in each of the 7 diseases, and used the different algorithms to predict the disease status of the held-out test individuals, as well as the algorithms' majority vote. We summarize our predictive power using the area under the receiver operating curve (AUC) value, as it is widely used to assess predictive performance in a case-control scenario. Intuitively, the AUC can be thought of as the probability that a predictor will correctly classify a pair of samples, one positive and one negative. A perfect predictor therefore has an AUC of 1, whereas a random predictor has an AUC of 0.5.

**Figure 3:**
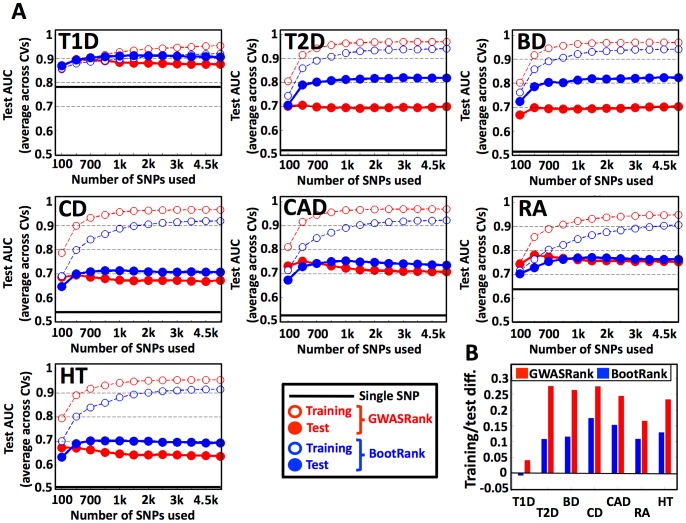
BootRank improves disease risk prediction for held-out test individuals. (a) For each disease (T1D, Type 1 diabetes; T2D, Type 2 diabetes; CD, Crohn's disease; CAD, coronary artery disease; BD, bipolar disorder; RA, rheumatoid arthritis; HT, hypertension), shown are the training (empty circles) and test (filled circles) AUC values as a function of different numbers of SNPs used in the model (x-axis) when employing either GWASRank (red) or BootRank (blue) to rank SNPs. (b) The mean difference between training AUC and test AUC is shown for all diseases for GWASRank (red) or BootRank (blue).

Notably, we found that in three diseases, T2D, BD, and HT, using BootRank compared to GWASRank significantly increased the test AUC from 0.69, 0.68 and 0.65 to 0.82, 0.83 and 0.68, respectively, and classification accuracy by ∼7% on average (**Tables 1** **and S10, 11,** **Figures 3** **and S1, S2, S3, S4, S5, S6, S7**). In the remaining 4 diseases (T1D, CD, CAD, and RA), using BootRank was not significantly beneficial to using GWASRank. Moreover, these three diseases for which BootRank improved the performance the most (i.e., T2D, BD and HT) were recently found to have an underestimated value of heritability (h^2^), probably because they are mainly affected by variants that have a low minimum allele frequency (MAF) [22]. This result suggests that BootRank is especially beneficial in cases were the underlying SNPs that affect the disease are poorly tagged or have low MAF.

**Table 1:** Prediction performance for WTCCC data on test data.

Disease/method	T1D	T2D	BD	CD	CAD	RA	HT
**BootRank + Majority**	**0.90**	**0.82**	**0.83**	**0.70**	**0.72**	**0.74**	**0.68**
**GWASRank + Majority**	**0.88**	**0.69**	**0.68**	**0.67**	**0.72**	**0.75**	**0.65**
LO, AC [15]	0.75	0.6	0.67	0.63	0.6	0.67	0.61
SVM [67]	0.82					0.71	
GWASelect [24]	0.79						
SVM, LR [63]	0.89						
Forward ROC [64]						0.71	
LR, SVM, RF, BN [65]			0.56				
Elastic-net [16]				0.64			
LR, AC, SVM [66]					0.6		

Shown are the AUC values obtained by different studies across the seven diseases in the WTCCC dataset. The reported AUCs were calculated only for test individuals. For each study, we took the best AUC reported for each disease, and missing diseases were left blank.

Diseases: T1D, Type 1 diabetes; T2D, Type 2 diabetes; CD, Crohn's disease; CAD, coronary artery disease; BD, bipolar disorder; RA, rheumatoid arthritis; HT, hypertension. Algorithms: SVM, support vector machine; LR, logistic regression; AC, allele count; RF, random forest; LO, log odds; BN, Bayesian networks.

We also found that in all cases, predictors based on multiple SNPs ranked by either GWASRank or BootRank had better test AUCs than a predictor based on the best single SNP in the training set, and that in all cases BootRank significantly decreased the over-fitting of the model, as seen by the lower difference between training and test results (**Figure 3b**).

Next, we compared the performance of the 7 different classification algorithms as well as their majority vote in terms of their AUC values, precision-recall, and accuracy (**Tables 2** **, S8, S9, S10, S11**). We found that for each disease, there is one algorithm that performs best, but that overall, the combined majority vote either outperforms individual algorithms or is very close in performance to the best one, encouraging the use of such multi-algorithm approaches to prediction of disease risk.

**Table 2:** Mean test AUC for different algorithms using BootRank.

Disease/algorithm	T1D	T2D	BD	CD	CAD	RA	HT
Support vector machine (SVM)	0.90	0.76	0.78	0.64	0.63	0.71	0.61
Random forest (RF)	0.88	0.76	0.77	0.65	0.68	0.71	0.64
Regularized logistic regression (RLR)	0.91	0.77	0.76	0.696	0.71	0.78	0.68
Naïve Bayes (NB)	0.77	0.83	0.83	0.67	0.72	0.71	0.68
Allele count (AC)	0.80	0.79	0.80	0.63	0.59	0.65	0.61
Log Odds (LO)	0.81	0.81	0.81	0.699	0.69	0.71	0.67
Robust adaboost (RAB)	0.89	0.78	0.78	0.695	0.75	0.75	0.71
Majority (all algorithms)	0.90	0.82	0.83	0.70	0.72	0.74	0.68
4-Majority (only RF, RLR, NB and RAB)	0.91	0.82	0.82	0.71	0.75	0.77	0.70

Shown are the average AUC values for test individuals for the different algorithms when using BootRank, or when combining all 7 algorithms (Majority), or only 4 algorithms (4-Majority).

Finally, we sought to compare our predictive power with that reported in other studies. Since different datasets have different properties, such as number of cases and controls, genotyping density and sampling biases, we only considered for this comparison predictions made on the WTCCC data using a strict cross-validation (CV) training and test scheme (i.e., where as we did here, the test data was not used during the training process) [15], [16], [24], [63]–[67]. We found that in all of these cases, our approach achieved better predictions (**Table 1**). We note that we excluded methods such as [36], in which the ranking of SNPs was partly done on the entire dataset before splitting into training and test, because such a setting makes use of the held-out test data during the training phase.

Taken together, we show that multi-SNP models can significantly outperform single-SNP models in disease risk prediction, and that BootRank improves the performance and robustness of the predictions over GWASRank. In addition, we show that using BootRank with a majority vote of several algorithms achieves higher AUC on test data compared to previous reports in the literature.

## Discussion

Predicting the risk of individuals to develop a disease given their genetic sequence is a desirable goal, yet the current ability to make such predictions is relatively poor. Since they highly depend on the initial ranking of SNPs that is given to them as input, one common limitation of current methods is the dependence of this input set of SNPs on noise or on the specific choice of data.

Here, we presented *BootRank*, a SNP ranking method based on bootstrapping, and applied it to the Wellcome Trust Case Control Consortium (WTCCC) data [21] consisting of thousands of individuals suffering from seven different diseases. We first showed that using BootRank results in a more robust SNP ranking compared to using the GWAS p-value (*GWASRank*), and in more significantly enriched pathways for the different diseases. Moreover, the pathways detected have considerable support in the literature as being involved in the different diseases, validating the biological merit of ranking SNPs by BootRank.

Next, we showed that BootRank could improve disease status prediction in held-out test individuals, by comparing the performance of an approach that uses a single SNP, to approaches that use multiple SNPs ranked by BootRank or by GWASRank. It is important to note, that we are not trying to identify the list of “truly” associated SNPs. In a sense, we do not believe that such a list exists, since many SNP can have a very small effect, or be context specific (e.g., affect only if another SNP is present), or be environment-dependent (e.g., affect only if a certain virus is attacking the cells). Therefore, BootRank is not a feature-selection algorithm *per se*, but rather its goal is to improve the ranking of SNPs with respect to a disease, and let the classification algorithm use the data at hand to do the final feature selection and model building. For the multi-SNP models, we compared seven different classification algorithms as well as their majority vote. We found that using both multi-SNP models outperform the single-SNP model, and that BootRank significantly improves the test AUC in 3/7 of diseases (T2D, BD and HT, by 0.03–0.15) compared to GWASRank. In addition, we showed that using BootRank with the majority vote of all seven algorithms outperforms previous disease status prediction values of test individuals in the WTCCC data.

Although the improvement over existing publications is large in some cases (e.g., Type 2 diabetes improves from 0.6 to 0.82 AUC), in Type 1 diabetes (T1D) we were not able to improve significantly over existing work and the test AUC remained ∼0.9, suggesting that in T1D we are perhaps approaching the limit of predictive power solely from SNPs. The rest of the variance among individuals could be attributed either to other genetic alterations such as copy number or structural variations (CNVs and SVs, respectively), epigenetic differences such as methylation, or environmental factors such as nutrition or maternal effects.

In the other diseases where our test AUCs ranged from ∼0.7 to ∼0.8, the results are not yet clinically relevant, especially since the propensity of disease cases in the population is low, and thus having low specificity would generate many false positives. Clearly, for these diseases, more could be done in order to improve performance. Adding other genetic variants such as CNVs and SVs could boost performance, as can integrating existing biological knowledge such as pathway information, or protein-protein interaction networks. We note that testing our method on an external dataset to see how well it generalizes would be very beneficial, but this is currently beyond the scope of this work.

The ability to predict disease risk across individuals could transform human health by directing life-style changes among high-risk individuals or by helping early diagnosis by promoting periodical screenings. The ideal risk predictor would make use of both genetic and epigenetic variants, as well as take into account known biological mechanisms and environmental factors, and will enable individuals to appreciate their different risks, aiding them to make the right decisions regarding their health.

## Methods

### Genotype and phenotype datasets

All genotype and phenotype (disease state) data was obtained from the Wellcome Trust Case Control Consortium (WTCCC) [21], consisting of 7 different disease sets, with ∼2000 cases for each disease, and a shared set of ∼1500 control individuals. All SNPs that were removed by the original publication due to bad quality, deviation from Hardy-Weinberg equilibrium or bad clustering were removed in this study as well. No correction for family structure was applied to the data.

### Computing GWAS p-values for SNPs

For each set of cases and controls of a certain disease, 4 different p-values were calculated for each SNP corresponding to the 4 possible genetic models:

1. General: Chi-square test for a 3×2 contingency table, where each genotype (i.e., common/common, common/rare and rare/rare) is counted separately. This test has 2 degrees of freedom.
2. Dominant: Chi-square test for a 2×2 contingency table, where common/rare and rare/rare are counted together. This test has 1 degree of freedom.
3. Recessive: Chi-square test for a 2×2 contingency table, where common/common and common/rare are counted together. This test has 1 degree of freedom.
4. Additive: Chi-square test for a 2×2 contingency table, where alleles are counted instead of genotypes (e.g., each genotype is counted as two alleles). This test has 1 degree of freedom.

The best (lowest) p-value out of the 4 was assigned to each SNP as the GWAS p-value.

### Bootstrap ranking of SNPs

For a given training set of *N* individuals, 100 bootstrap samples were generated by randomly selecting *N* individuals with replacement. For each such sample, GWAS p-values were calculated for all SNPs, and the corresponding ranking was recorded (*GWASRank*). The final bootstrap ranking (*BootRank*) is based on the median rank each SNP achieved across the 100 bootstrap samples. The BootRank code was written in-house and has been made freely available for academic use in the following website: http://genie.weizmann.ac.il/pubs/BootRank/.

### Calculating average fraction of top-SNPs overlap between different cross-validation partitions

For a given 5-fold cross-validation (CV) partition into training and test, a SNP ranking was calculated (by either GWASRank or BootRank), and a number of top-SNPs were selected (e.g., top 1000) as the top-SNPs list for this partition. As a 5-fold CV has 5 such partitions, 5 lists of top-SNPs were calculated. Next, for each pair of partitions, the fraction of overlapping top-SNPs was calculated (i.e., how many top-SNPs they share out of a 1000), and the average across the 10 possible pairs was recorded. This procedure was repeated 10 times to remove possible biases from a specific CV partition, and the overall average of the 10 repeats was reported.

### Testing for KEGG pathway enrichments for the different diseases

For each disease, we created 10 repeats of 5-fold cross-validation sets, resulting overall in 50 training sets. For each training set, we first computed a SNP ranking (by either GWASRank or BootRank). Next, we converted the SNP ranking to a gene ranking, by assigning each gene the best rank obtained by a SNP that resides within it or within its flanking 5 kb region. For a given gene ranking, we computed enrichment p-values for all KEGG [31], [32] pathways by using the Wilcoxon rank-sum test using an in-house script. Intuitively, testing whether genes belonging to a pathway appear more at the top of the ranked list than expected by chance, by comparing the sum of their ranks to the sum of ranks of a random set of genes of equal size. A pathway was defined as significantly enriched in the disease if it passed an enrichment p-value of P<0.05 in at least 45/50 (90%) of the CV training sets.

### Predicting disease risk using different classification algorithms

For each disease cross-validation partition, we used the training set to produce a SNP ranking (by either GWASRank or BootRank). Next, we selected some top number of SNPs (e.g., 1000), and used these SNPs as input for one of seven classification algorithms: (1) Random forest (RF) [60]; (2) Regularized logistic regression (RLR) [61]; (3) A support vector machine (SVM) [62]; (4) Naïve Bayes (NB); (5) Robust adaboost (RAB); (6) Allele count (AC); and (7) Log odds (LO). Running the algorithms was done by either using built-in functions in MATLAB (e.g., Adaboost), or by coding the algorithm in MATLAB ourselves. After learning the discriminative model from training examples, disease risk was predicted for the held-out test individuals (i.e., not a binary prediction but rather a continuous one), and this ranking of test individuals (i.e., from most likely to have the disease to least likely) was used to compute the area under the receiver operating characteristic curve (AUC value).

### Parameters used or learned for the different classification algorithms

In all of the classification algorithms we set the different parameters based only on the training data.

In Random forest, we use an in-house implementation, and set the number of weak predictors to be 500, as we found that the AUC appears to converge around that point. For the number of random features selected at each node in the tree, we used the original recommendation of Breiman [60] as the square root of the total number of features.

In the Regularized logistic regression we use the GLMNET implementation [61], and set the penalty parameter using an internal cross-validation on the training data, where we a subset of the training is set aside as validation and the best penalty is chosen by the validation set. Once the penalty is chosen, we use it to learn the feature weights on the whole training set.

In the support vector machine we use the Radial basis function (RBF) kernel in all cases, and set the penalty parameter C to 1 and the gamma parameter to 1 over the number of features in the model, which are all the default values in the implementation we used (LIBSVM [62]).

In Naïve Bayes we use the Matlab implementation, and set the distribution type as Multivariate multinomial (since the data is discrete) and the prior to be empirical.

In Robust adaboost we use the Matlab implementation, with 500 learning cycles and trees as weak learners.

In Allele count and Log odds we use an in-house implementation and there are no fitted parameters.

### Combining predictions of different algorithms to form a majority vote

For a given cross-validation partition, the predicted rankings of held-out test individuals (i.e., from most likely to have the disease to least likely) were calculated for all algorithms. Next, the disease-risk rankings were combined by assigning each test individual the median ranking obtained across the different algorithms.

## Supporting Information

Figure S1 **BootRank improves disease risk prediction for held-out test individuals using the Random forest algorithm.** For each disease (T1D, Type 1 diabetes; T2D, Type 2 diabetes; CD, Crohn's disease; CAD, coronary artery disease; BD, bipolar disorder; RA, rheumatoid arthritis; HT, hypertension), shown are the training (empty circles) and test (filled circles) AUC values as a function of different numbers of SNPs used in the model (x-axis) when employing either GWASRank (red) or BootRank (blue) to rank SNPs.
(TIFF)

Figure S2 **BootRank improves disease risk prediction for held-out test individuals using the Support vector machine algorithm.** For each disease (T1D, Type 1 diabetes; T2D, Type 2 diabetes; CD, Crohn's disease; CAD, coronary artery disease; BD, bipolar disorder; RA, rheumatoid arthritis; HT, hypertension), shown are the training (empty circles) and test (filled circles) AUC values as a function of different numbers of SNPs used in the model (x-axis) when employing either GWASRank (red) or BootRank (blue) to rank SNPs.
(TIFF)

Figure S3 **BootRank improves disease risk prediction for held-out test individuals using a regularized Logistic regression algorithm.** For each disease (T1D, Type 1 diabetes; T2D, Type 2 diabetes; CD, Crohn's disease; CAD, coronary artery disease; BD, bipolar disorder; RA, rheumatoid arthritis; HT, hypertension), shown are the training (empty circles) and test (filled circles) AUC values as a function of different numbers of SNPs used in the model (x-axis) when employing either GWASRank (red) or BootRank (blue) to rank SNPs.
(TIFF)

Figure S4 **BootRank improves disease risk prediction for held-out test individuals using the Naïve Bayes algorithm.** For each disease (T1D, Type 1 diabetes; T2D, Type 2 diabetes; CD, Crohn's disease; CAD, coronary artery disease; BD, bipolar disorder; RA, rheumatoid arthritis; HT, hypertension), shown are the training (empty circles) and test (filled circles) AUC values as a function of different numbers of SNPs used in the model (x-axis) when employing either GWASRank (red) or BootRank (blue) to rank SNPs.
(TIFF)

Figure S5 **BootRank improves disease risk prediction for held-out test individuals using the Allele count algorithm.** For each disease (T1D, Type 1 diabetes; T2D, Type 2 diabetes; CD, Crohn's disease; CAD, coronary artery disease; BD, bipolar disorder; RA, rheumatoid arthritis; HT, hypertension), shown are the training (empty circles) and test (filled circles) AUC values as a function of different numbers of SNPs used in the model (x-axis) when employing either GWASRank (red) or BootRank (blue) to rank SNPs.
(TIFF)

Figure S6 **BootRank improves disease risk prediction for held-out test individuals using the Log odds algorithm.** For each disease (T1D, Type 1 diabetes; T2D, Type 2 diabetes; CD, Crohn's disease; CAD, coronary artery disease; BD, bipolar disorder; RA, rheumatoid arthritis; HT, hypertension), shown are the training (empty circles) and test (filled circles) AUC values as a function of different numbers of SNPs used in the model (x-axis) when employing either GWASRank (red) or BootRank (blue) to rank SNPs.
(TIFF)

Figure S7 **BootRank improves disease risk prediction for held-out test individuals using a Robust Adaboost algorithm.** For each disease (T1D, Type 1 diabetes; T2D, Type 2 diabetes; CD, Crohn's disease; CAD, coronary artery disease; BD, bipolar disorder; RA, rheumatoid arthritis; HT, hypertension), shown are the training (empty circles) and test (filled circles) AUC values as a function of different numbers of SNPs used in the model (x-axis) when employing either GWASRank (red) or BootRank (blue) to rank SNPs.
(TIFF)

Figure S8 **Fraction of intersection of filtered SNPs lists between different cross-validation partitions for different subsets of the data.** For each disease (T1D, Type 1 diabetes; T2D, Type 2 diabetes; CD, Crohn's disease; CAD, coronary artery disease; BD, bipolar disorder; RA, rheumatoid arthritis; HT, hypertension), shown is the mean fraction (y-axis) of top SNPs shared between training sets from different cross-validations when ranking SNPs by GWASRank (red) or BootRank (blue), for different sizes of subsets of the data (i.e., 25%, 50%, 75% and 100%, marked by different symbols). The x-axis shows the number of SNPs that were selected as top SNPs from the SNP ranking.
(TIFF)

Table S1 **T1D differential pathway enrichment for BootRank and GWASRank.** Columns are: KEGG pathway ID, KEGG pathway name, median p-value for GWASRank (missing if non-significant), median p-value for BootRank (missing if non-significant), Supporting reference in the literature.
(DOCX)

Table S2 **T2D differential pathway enrichment for BootRank and GWASRank.** Columns are: KEGG pathway ID, KEGG pathway name, median p-value for GWASRank (missing if non-significant), median p-value for BootRank (missing if non-significant), Supporting reference in the literature.
(DOCX)

Table S3 **BD differential pathway enrichment for BootRank and GWASRank.** Columns are: KEGG pathway ID, KEGG pathway name, median p-value for GWASRank (missing if non-significant), median p-value for BootRank (missing if non-significant), Supporting reference in the literature.
(DOCX)

Table S4 **CAD differential pathway enrichment for BootRank and GWASRank.** Columns are: KEGG pathway ID, KEGG pathway name, median p-value for GWASRank (missing if non-significant), median p-value for BootRank (missing if non-significant), Supporting reference in the literature.
(DOCX)

Table S5 **HT differential pathway enrichment for BootRank and GWASRank.** Columns are: KEGG pathway ID, KEGG pathway name, median p-value for GWASRank (missing if non-significant), median p-value for BootRank (missing if non-significant), Supporting reference in the literature.
(DOCX)

Table S6 **CD differential pathway enrichment for BootRank and GWASRank.** Columns are: KEGG pathway ID, KEGG pathway name, median p-value for GWASRank (missing if non-significant), median p-value for BootRank (missing if non-significant), Supporting reference in the literature.
(DOCX)

Table S7 **RA differential pathway enrichment for BootRank and GWASRank.** Columns are: KEGG pathway ID, KEGG pathway name, median p-value for GWASRank (missing if non-significant), median p-value for BootRank (missing if non-significant), Supporting reference in the literature.
(DOCX)

Table S8 **Mean test AUC-PR for different algorithms using BootRank.** Shown are the average AUC values for the Precision-Recall (PR) curve for test individuals for the different algorithms when using BootRank, or when combining all 7 algorithms (Majority), or only 4 algorithms (4-Majority). The best single algorithm for each disease is highlighted in gray.
(DOCX)

Table S9 **Mean test AUC-PR for different algorithms using GWASRank.** Shown are the average AUC values for the Precision-Recall (PR) curve for test individuals for the different algorithms when using GWASRank, or when combining all 7 algorithms (Majority), or only 4 algorithms (4-Majority). The best single algorithm for each disease is highlighted in gray.
(DOCX)

Table S10 **Mean test classification accuracy for different algorithms using BootRank.** Shown is the average classification accuracy for test individuals for the different algorithms when using BootRank, or when combining all 7 algorithms (Majority), or only 4 algorithms (4-Majority). The best single algorithm for each disease is highlighted in gray.
(DOCX)

Table S11 **Mean test classification accuracy for different algorithms using GWASRank.** Shown is the average classification accuracy for test individuals for the different algorithms when using GWASRank, or when combining all 7 algorithms (Majority), or only 4 algorithms (4-Majority). The best single algorithm for each disease is highlighted in gray.
(DOCX)
