# Supplementary figures and images for: Predicting Disease Risk Using Bootstrap Ranking and Classification Algorithms
Source: PLoS Comput Biol. 2013 Aug 22;9(8):e1003200. doi: 10.1371/journal.pcbi.1003200 (PMC3749941; doi:10.1371/journal.pcbi.1003200)

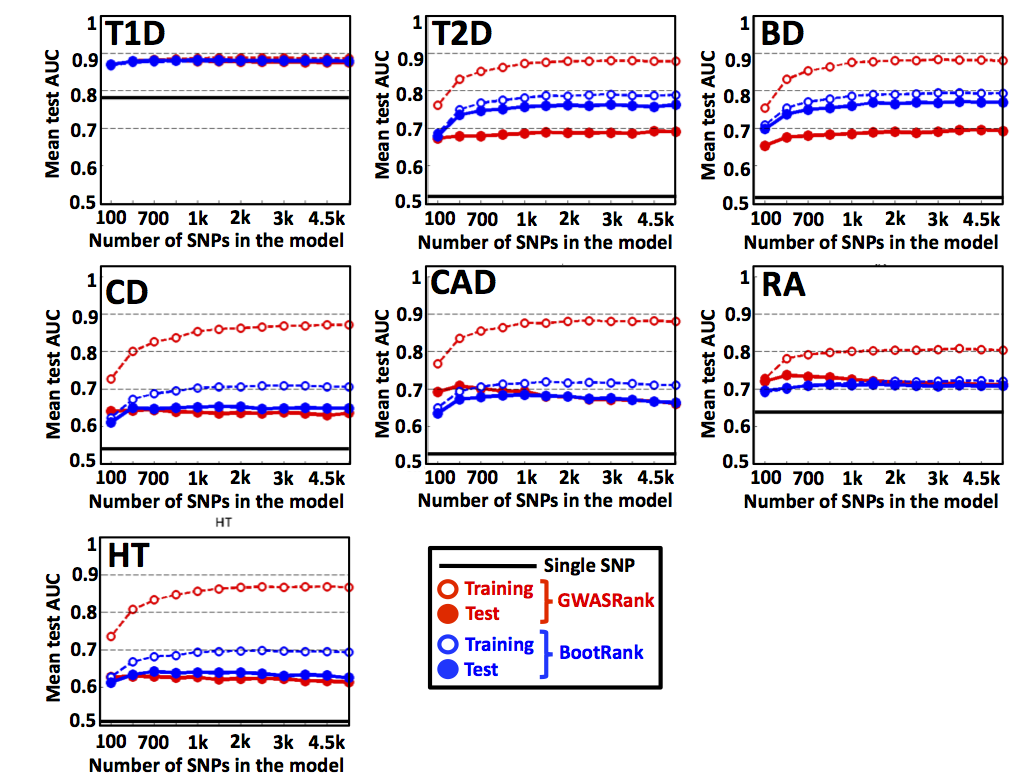

Supplement: Figure S1 — BootRank improves disease risk prediction for held-out test individuals using the Random forest algorithm. For each disease (T1D, Type 1 diabetes; T2D, Type 2 diabetes; CD, Crohn's disease; CAD, coronary artery disease; BD, bipolar disorder; RA, rheumatoid arthritis; HT, hypertension), shown are the training (empty circles) and test (filled circles) AUC values as a function of different numbers of SNPs used in the model (x-axis) when employing either GWASRank (red) or BootRank (blue) to rank SNPs. (TIFF) [file pcbi.1003200.s001.tiff]

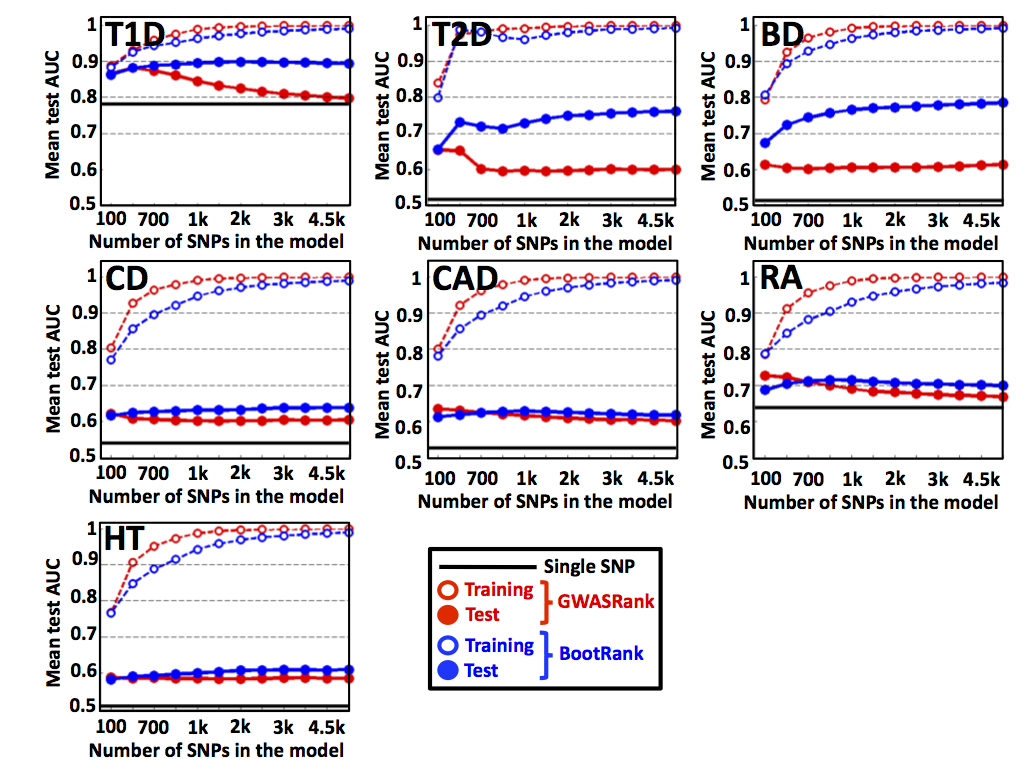

Supplement: Figure S2 — BootRank improves disease risk prediction for held-out test individuals using the Support vector machine algorithm. For each disease (T1D, Type 1 diabetes; T2D, Type 2 diabetes; CD, Crohn's disease; CAD, coronary artery disease; BD, bipolar disorder; RA, rheumatoid arthritis; HT, hypertension), shown are the training (empty circles) and test (filled circles) AUC values as a function of different numbers of SNPs used in the model (x-axis) when employing either GWASRank (red) or BootRank (blue) to rank SNPs. (TIFF) [file pcbi.1003200.s002.tiff]

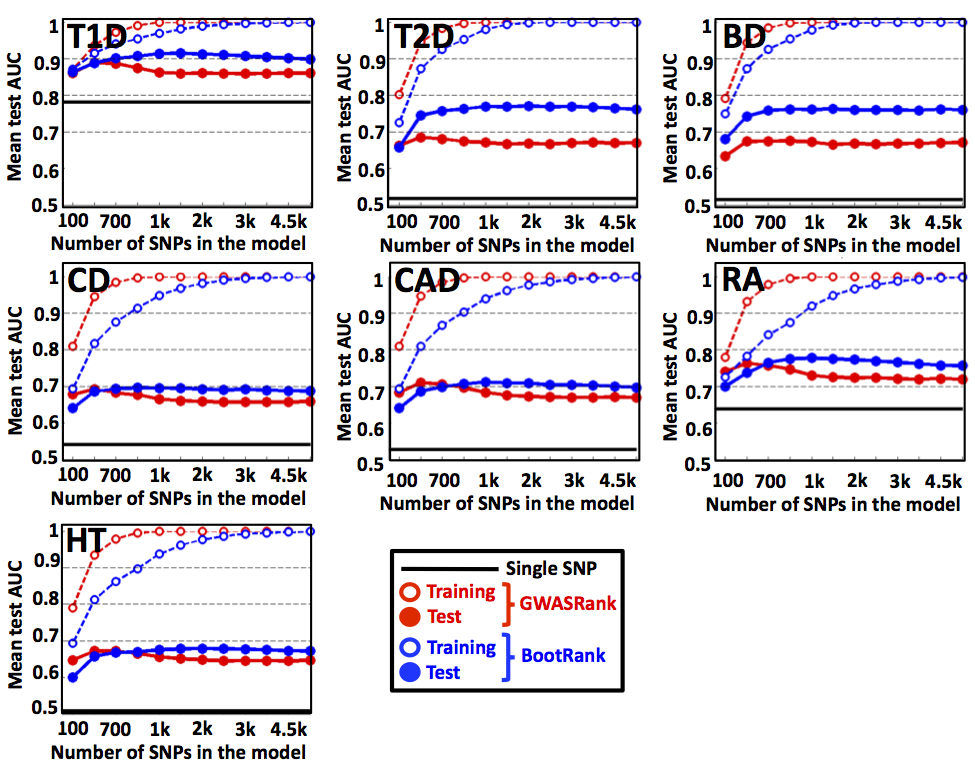

Supplement: Figure S3 — BootRank improves disease risk prediction for held-out test individuals using a regularized Logistic regression algorithm. For each disease (T1D, Type 1 diabetes; T2D, Type 2 diabetes; CD, Crohn's disease; CAD, coronary artery disease; BD, bipolar disorder; RA, rheumatoid arthritis; HT, hypertension), shown are the training (empty circles) and test (filled circles) AUC values as a function of different numbers of SNPs used in the model (x-axis) when employing either GWASRank (red) or BootRank (blue) to rank SNPs. (TIFF) [file pcbi.1003200.s003.tiff]

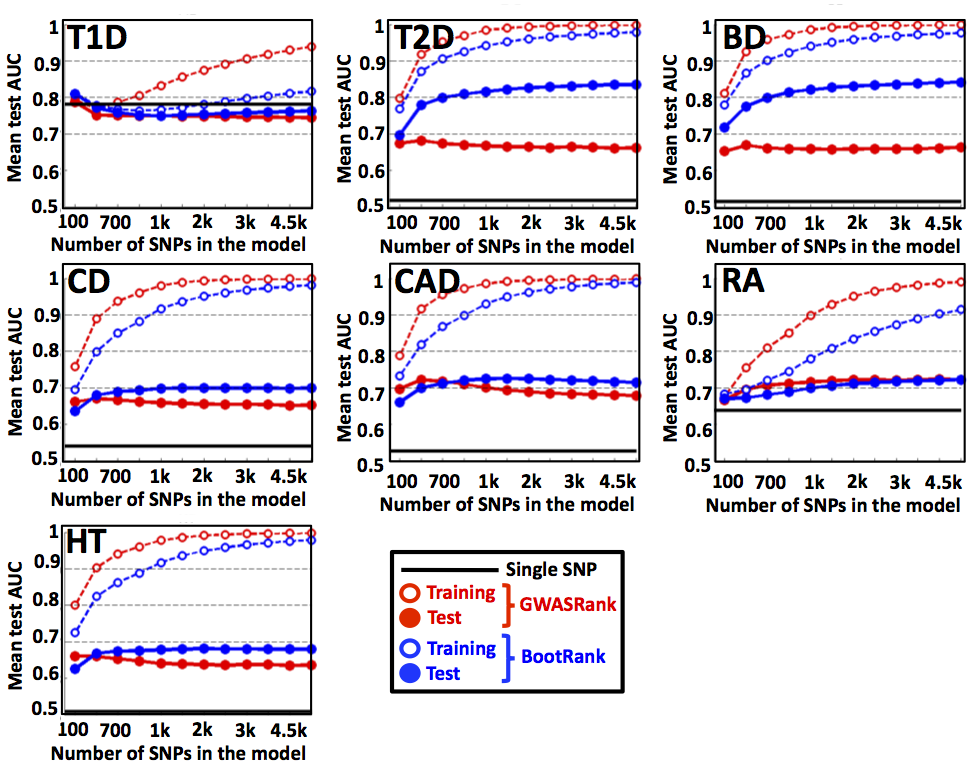

Supplement: Figure S4 — BootRank improves disease risk prediction for held-out test individuals using the Naïve Bayes algorithm. For each disease (T1D, Type 1 diabetes; T2D, Type 2 diabetes; CD, Crohn's disease; CAD, coronary artery disease; BD, bipolar disorder; RA, rheumatoid arthritis; HT, hypertension), shown are the training (empty circles) and test (filled circles) AUC values as a function of different numbers of SNPs used in the model (x-axis) when employing either GWASRank (red) or BootRank (blue) to rank SNPs. (TIFF) [file pcbi.1003200.s004.tiff]

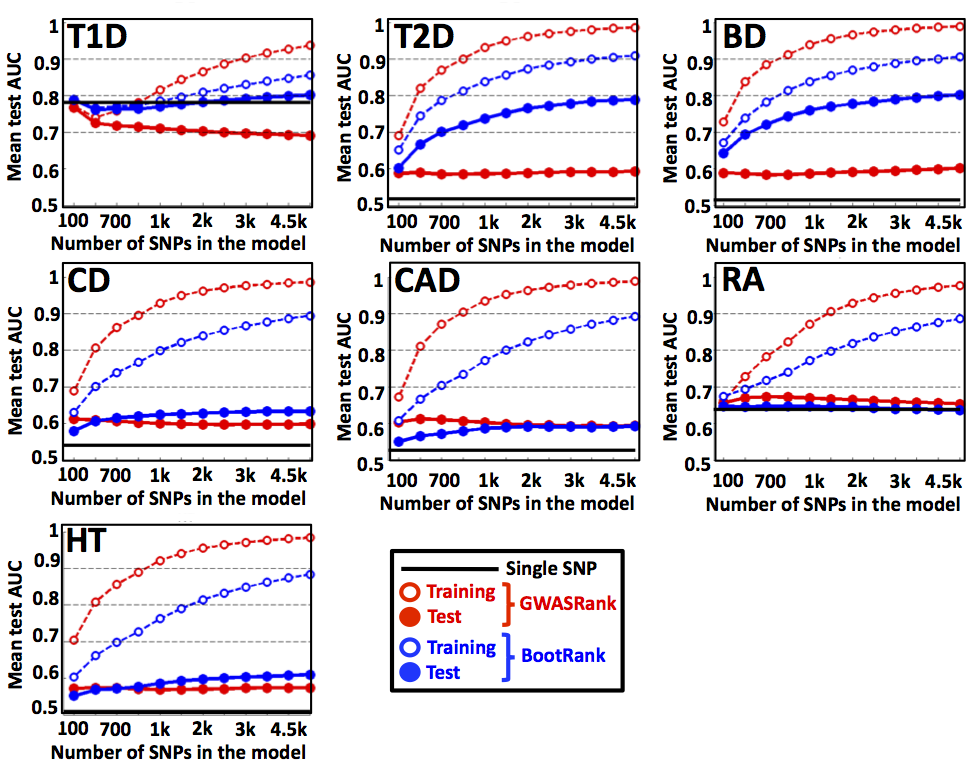

Supplement: Figure S5 — BootRank improves disease risk prediction for held-out test individuals using the Allele count algorithm. For each disease (T1D, Type 1 diabetes; T2D, Type 2 diabetes; CD, Crohn's disease; CAD, coronary artery disease; BD, bipolar disorder; RA, rheumatoid arthritis; HT, hypertension), shown are the training (empty circles) and test (filled circles) AUC values as a function of different numbers of SNPs used in the model (x-axis) when employing either GWASRank (red) or BootRank (blue) to rank SNPs. (TIFF) [file pcbi.1003200.s005.tiff]

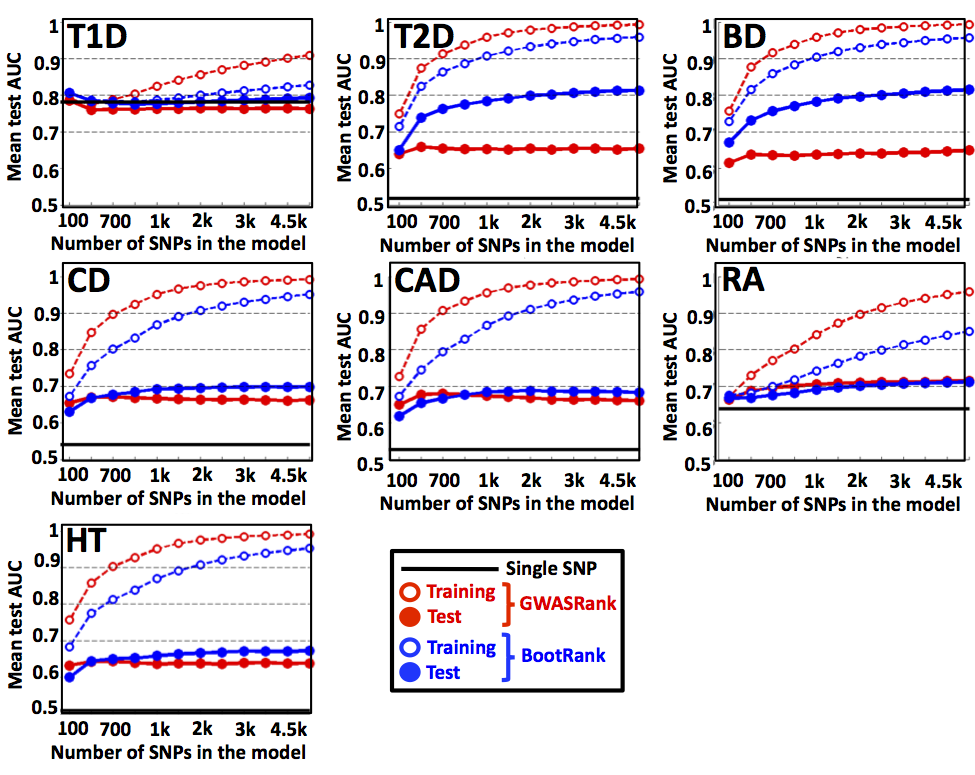

Supplement: Figure S6 — BootRank improves disease risk prediction for held-out test individuals using the Log odds algorithm. For each disease (T1D, Type 1 diabetes; T2D, Type 2 diabetes; CD, Crohn's disease; CAD, coronary artery disease; BD, bipolar disorder; RA, rheumatoid arthritis; HT, hypertension), shown are the training (empty circles) and test (filled circles) AUC values as a function of different numbers of SNPs used in the model (x-axis) when employing either GWASRank (red) or BootRank (blue) to rank SNPs. (TIFF) [file pcbi.1003200.s006.tiff]

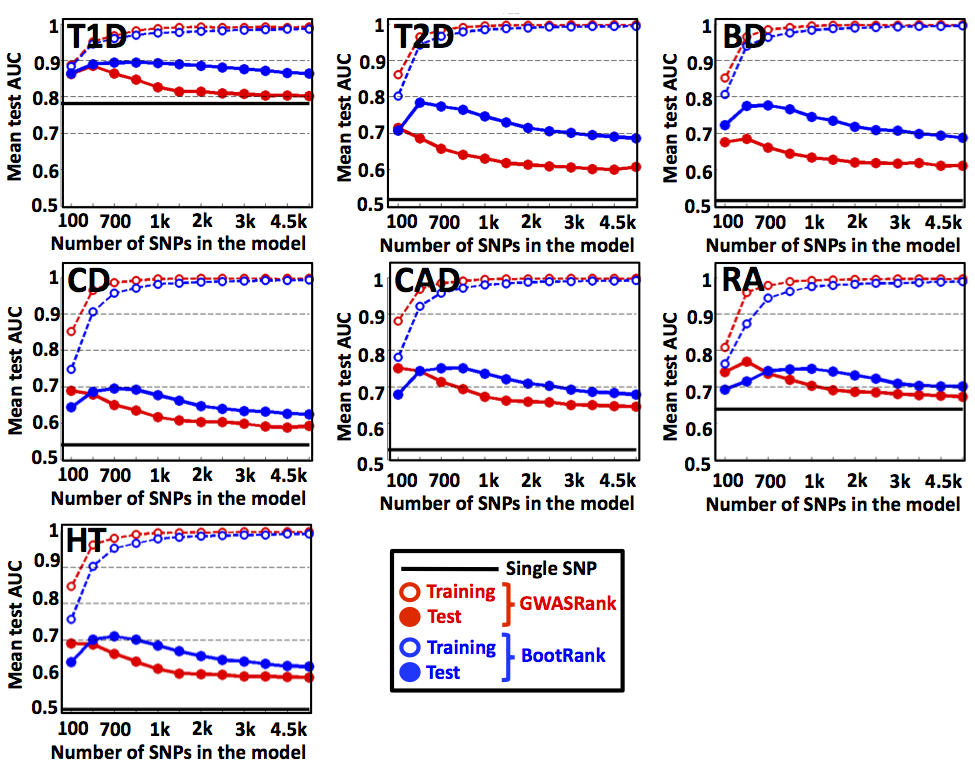

Supplement: Figure S7 — BootRank improves disease risk prediction for held-out test individuals using a Robust Adaboost algorithm. For each disease (T1D, Type 1 diabetes; T2D, Type 2 diabetes; CD, Crohn's disease; CAD, coronary artery disease; BD, bipolar disorder; RA, rheumatoid arthritis; HT, hypertension), shown are the training (empty circles) and test (filled circles) AUC values as a function of different numbers of SNPs used in the model (x-axis) when employing either GWASRank (red) or BootRank (blue) to rank SNPs. (TIFF) [file pcbi.1003200.s007.tiff]

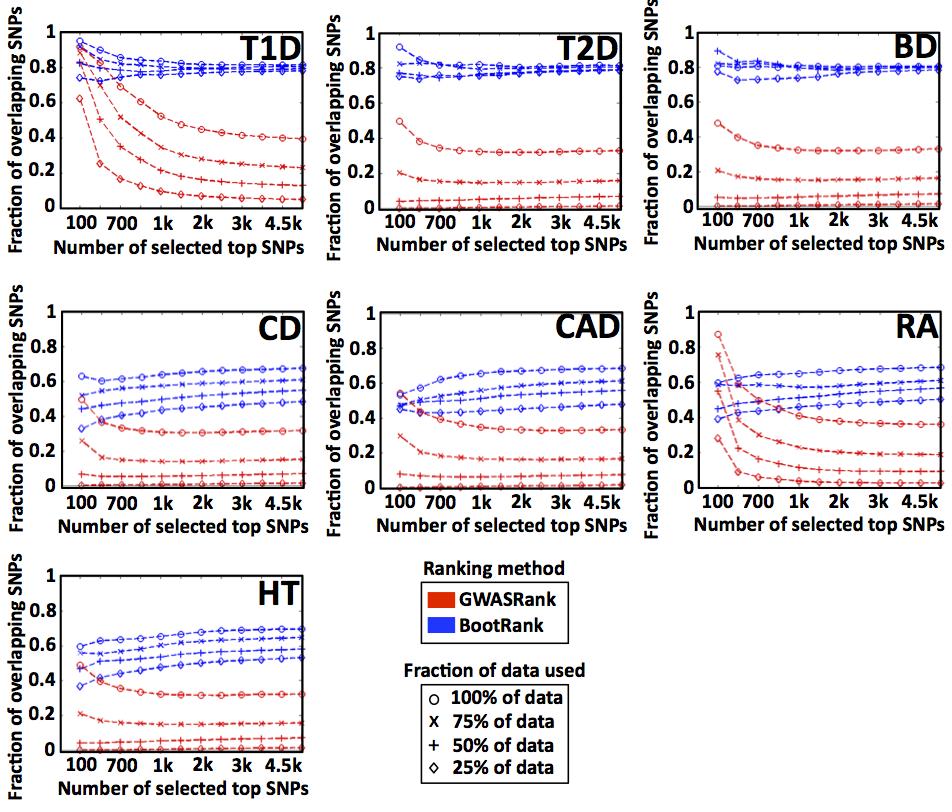

Supplement: Figure S8 — Fraction of intersection of filtered SNPs lists between different cross-validation partitions for different subsets of the data. For each disease (T1D, Type 1 diabetes; T2D, Type 2 diabetes; CD, Crohn's disease; CAD, coronary artery disease; BD, bipolar disorder; RA, rheumatoid arthritis; HT, hypertension), shown is the mean fraction (y-axis) of top SNPs shared between training sets from different cross-validations when ranking SNPs by GWASRank (red) or BootRank (blue), for different sizes of subsets of the data (i.e., 25%, 50%, 75% and 100%, marked by different symbols). The x-axis shows the number of SNPs that were selected as top SNPs from the SNP ranking. (TIFF) [file pcbi.1003200.s008.tiff]
