# Supplementary material for: Predicting Disease Risk Using Bootstrap Ranking and Classification Algorithms
Source: PLoS Comput Biol. 2013 Aug 22;9(8):e1003200. doi: 10.1371/journal.pcbi.1003200 (PMC3749941; doi:10.1371/journal.pcbi.1003200)
Supplement: Table S1 — T1D differential pathway enrichment for BootRank and GWASRank. Columns are: KEGG pathway ID, KEGG pathway name, median p-value for GWASRank (missing if non-significant), median p-value for BootRank (missing if non-significant), Supporting reference in the literature. (DOCX) [file pcbi.1003200.s009.docx]

| **Pathway ID** | **Pathway name** | **GWASRank** | **BootRank** | **Supporting reference** |
| --- | --- | --- | --- | --- |
| hsa00510 | N-Glycan biosynthesis | 0.00965 | - |  |
| hsa04612 | Antigen processing and presentation | 0.00291 | - |  |
| hsa00640 | Propanoate metabolism | - | 0.000644 |  |
| hsa04914 | Progesterone-mediated oocyte maturation | - | 0.00155 |  |
| hsa04010 | MAPK signaling pathway | - | 0.0016 | [36] |
| hsa05020 | Prion diseases | - | 0.00758 |  |
| hsa00290 | Valine, leucine and isoleucine biosynthesis | - | 0.00761 | [33] |
| hsa05214 | Glioma | - | 0.0129 |  |
| hsa04062 | Chemokine signaling pathway | - | 0.0138 | [34,35] |
| hsa04920 | Adipocytokine signaling pathway | - | 0.0211 |  |
| hsa04664 | Fc epsilon RI signaling pathway | - | 0.0221 |  |
| hsa04976 | Bile secretion | - | 0.0247 |  |
| hsa04722 | Neurotrophin signaling pathway | - | 0.03 |  |
| hsa04114 | Oocyte meiosis | - | 0.0327 |  |
| hsa00300 | Lysine biosynthesis | - | 0.0332 |  |
