# Supplementary material for: Predicting Disease Risk Using Bootstrap Ranking and Classification Algorithms
Source: PLoS Comput Biol. 2013 Aug 22;9(8):e1003200. doi: 10.1371/journal.pcbi.1003200 (PMC3749941; doi:10.1371/journal.pcbi.1003200)
Supplement: Table S2 — T2D differential pathway enrichment for BootRank and GWASRank. Columns are: KEGG pathway ID, KEGG pathway name, median p-value for GWASRank (missing if non-significant), median p-value for BootRank (missing if non-significant), Supporting reference in the literature. (DOCX) [file pcbi.1003200.s010.docx]

| **Pathway ID** | **Pathway name** | **GWASRank** | **BootRank** | **Supporting reference** |
| --- | --- | --- | --- | --- |
| hsa04662 | B cell receptor signaling pathway | 4.93E-03 | - |  |
| hsa04666 | Fc gamma R-mediated phagocytosis | 7.18E-03 | - |  |
| hsa05222 | Small cell lung cancer | 1.04E-02 | - |  |
| hsa00534 | Glycosaminoglycan biosynthesis - heparan sulfate | - | 2.68E-03 |  |
| hsa03430 | Mismatch repair | - | 4.64E-03 |  |
| hsa04114 | Oocyte meiosis | - | 6.60E-03 |  |
| hsa04320 | Dorso-ventral axis formation | - | 1.26E-02 |  |
| hsa04610 | Complement and coagulation cascades | - | 0.0278 | [41,42] |
| hsa00232 | Caffeine metabolism | - | 0.031 | [38-40] |
| hsa04930 | Type II diabetes mellitus | - | 0.0313 | [37] |
| hsa00592 | alpha-Linolenic acid metabolism | - | 0.0326 | [43] |
| hsa00524 | Butirosin and neomycin biosynthesis | - | 0.0336 |  |
| hsa01040 | Biosynthesis of unsaturated fatty acids | - | 0.0338 |  |
