# Supplementary material for: Predicting Disease Risk Using Bootstrap Ranking and Classification Algorithms
Source: PLoS Comput Biol. 2013 Aug 22;9(8):e1003200. doi: 10.1371/journal.pcbi.1003200 (PMC3749941; doi:10.1371/journal.pcbi.1003200)
Supplement: Table S3 — BD differential pathway enrichment for BootRank and GWASRank. Columns are: KEGG pathway ID, KEGG pathway name, median p-value for GWASRank (missing if non-significant), median p-value for BootRank (missing if non-significant), Supporting reference in the literature. (DOCX) [file pcbi.1003200.s011.docx]

| **Pathway ID** | **Pathway name** | **GWASRank** | **BootRank** | **Supporting reference** |
| --- | --- | --- | --- | --- |
| hsa00053 | Ascorbate and aldarate metabolism | 0.000155 | - |  |
| hsa00140 | Steroid hormone biosynthesis | 9.23E-03 | - |  |
| hsa00500 | Starch and sucrose metabolism | 7.48E-03 | - |  |
| hsa00860 | Porphyrin and chlorophyll metabolism | 0.000629 | - |  |
| hsa00982 | Drug metabolism - cytochrome P450 | 0.00454 | - |  |
| hsa00983 | Drug metabolism - other enzymes | 0.0031 | - |  |
| hsa04662 | B cell receptor signaling pathway | - | 6.65E-03 |  |
| hsa00640 | Propanoate metabolism | - | 7.76E-03 | [45] |
| hsa00300 | Lysine biosynthesis | - | 1.86E-02 |  |
| hsa04080 | Neuroactive ligand-receptor interaction | - | 0.0259 | [44] |
