# Supplementary material for: Predicting Disease Risk Using Bootstrap Ranking and Classification Algorithms
Source: PLoS Comput Biol. 2013 Aug 22;9(8):e1003200. doi: 10.1371/journal.pcbi.1003200 (PMC3749941; doi:10.1371/journal.pcbi.1003200)
Supplement: Table S4 — CAD differential pathway enrichment for BootRank and GWASRank. Columns are: KEGG pathway ID, KEGG pathway name, median p-value for GWASRank (missing if non-significant), median p-value for BootRank (missing if non-significant), Supporting reference in the literature. (DOCX) [file pcbi.1003200.s012.docx]

| **Pathway ID** | **Pathway name** | **GWASRank** | **BootRank** | **Supporting reference** |
| --- | --- | --- | --- | --- |
| hsa00300 | Lysine biosynthesis | 9.00E-03 | - |  |
| hsa00562 | Inositol phosphate metabolism | 1.76E-02 | - |  |
| hsa05416 | Viral myocarditis | - | 0.00199 |  |
| hsa04930 | Type II diabetes mellitus | - | 0.00455 | [46,47] |
| hsa05210 | Colorectal cancer | - | 0.0133 | [48] |
| hsa04940 | Type I diabetes mellitus | - | 1.74E-02 |  |
| hsa05213 | Endometrial cancer | - | 1.82E-02 | [49] |
