# Supplementary material for: Predicting Disease Risk Using Bootstrap Ranking and Classification Algorithms
Source: PLoS Comput Biol. 2013 Aug 22;9(8):e1003200. doi: 10.1371/journal.pcbi.1003200 (PMC3749941; doi:10.1371/journal.pcbi.1003200)
Supplement: Table S5 — HT differential pathway enrichment for BootRank and GWASRank. Columns are: KEGG pathway ID, KEGG pathway name, median p-value for GWASRank (missing if non-significant), median p-value for BootRank (missing if non-significant), Supporting reference in the literature. (DOCX) [file pcbi.1003200.s013.docx]

| **Pathway ID** | **Pathway name** | **GWASRank** | **BootRank** | **Supporting reference** |
| --- | --- | --- | --- | --- |
| hsa04914 | Progesterone-mediated oocyte maturation | 5.93E-03 | - |  |
| hsa04976 | Bile secretion | 6.01E-03 | - |  |
| hsa05222 | Small cell lung cancer | 1.36E-02 | - |  |
| hsa05014 | Amyotrophic lateral sclerosis (ALS) | - | 6.72E-03 | [50] |
| hsa00565 | Ether lipid metabolism | - | 1.03E-02 | [51] |
| hsa00564 | Glycerophospholipid metabolism | - | 1.07E-02 |  |
| hsa00592 | alpha-Linolenic acid metabolism | - | 1.57E-02 |  |
| hsa05020 | Prion diseases | - | 2.33E-02 |  |
| hsa01040 | Biosynthesis of unsaturated fatty acids | - | 2.61E-02 |  |
| hsa00534 | Glycosaminoglycan biosynthesis - heparan sulfate | - | 2.62E-02 |  |
| hsa00760 | Nicotinate and nicotinamide metabolism | - | 2.75E-02 |  |
