# Supplementary material for: Predicting Disease Risk Using Bootstrap Ranking and Classification Algorithms
Source: PLoS Comput Biol. 2013 Aug 22;9(8):e1003200. doi: 10.1371/journal.pcbi.1003200 (PMC3749941; doi:10.1371/journal.pcbi.1003200)
Supplement: Table S6 — CD differential pathway enrichment for BootRank and GWASRank. Columns are: KEGG pathway ID, KEGG pathway name, median p-value for GWASRank (missing if non-significant), median p-value for BootRank (missing if non-significant), Supporting reference in the literature. (DOCX) [file pcbi.1003200.s014.docx]

| **Pathway ID** | **Pathway name** | **GWASRank** | **BootRank** | **Supporting reference** |
| --- | --- | --- | --- | --- |
| hsa04662 | B cell receptor signaling pathway | 0.015 | - |  |
| hsa04962 | Vasopressin-regulated water reabsorption | 0.0164 | - |  |
| hsa05145 | Toxoplasmosis | 0.00484 | - |  |
| hsa04080 | Neuroactive ligand-receptor interaction | - | 0.00721 | [56] |
| hsa00500 | Starch and sucrose metabolism | - | 0.0117 | [52,53] |
| hsa00770 | Pantothenate and CoA biosynthesis | - | 2.07E-02 | [54] |
| hsa04010 | MAPK signaling pathway | - | 2.18E-02 | [55] |
