# Supplementary material for: Predicting Disease Risk Using Bootstrap Ranking and Classification Algorithms
Source: PLoS Comput Biol. 2013 Aug 22;9(8):e1003200. doi: 10.1371/journal.pcbi.1003200 (PMC3749941; doi:10.1371/journal.pcbi.1003200)
Supplement: Table S7 — RA differential pathway enrichment for BootRank and GWASRank. Columns are: KEGG pathway ID, KEGG pathway name, median p-value for GWASRank (missing if non-significant), median p-value for BootRank (missing if non-significant), Supporting reference in the literature. (DOCX) [file pcbi.1003200.s015.docx]

| **Pathway ID** | **Pathway name** | **GWASRank** | **BootRank** | **Supporting reference** |
| --- | --- | --- | --- | --- |
| hsa04010 | MAPK signaling pathway | 0.00227 | - |  |
| hsa04145 | Phagosome | 0.00326 | - |  |
| hsa04930 | Type II diabetes mellitus | 0.0087 | - |  |
| hsa04940 | Type I diabetes mellitus | 2.64E-05 | - |  |
| hsa05140 | Leishmaniasis | 0.000331 | - |  |
| hsa05145 | Toxoplasmosis | 1.48E-05 | - |  |
| hsa05150 | Staphylococcus aureus infection | 0.0123 | - |  |
| hsa05222 | Small cell lung cancer | 0.00299 | - |  |
| hsa05320 | Autoimmune thyroid disease | 0.00196 | - |  |
| hsa05330 | Allograft rejection | 4.45E-05 | - |  |
| hsa05332 | Graft-versus-host disease | 0.00218 | - |  |
| hsa05416 | Viral myocarditis | 3.78E-05 | - |  |
| hsa04916 | Melanogenesis | - | 1.79E-05 | [57] |
| hsa00982 | Drug metabolism - cytochrome P450 | - | 0.000904 |  |
| hsa00980 | Metabolism of xenobiotics by cytochrome P450 | - | 0.00109 |  |
| hsa04920 | Adipocytokine signaling pathway | - | 0.00126 |  |
| hsa00830 | Retinol metabolism | - | 0.00278 |  |
| hsa04310 | Wnt signaling pathway | - | 0.00481 | [58] |
| hsa04976 | Bile secretion | - | 0.00933 |  |
| hsa01040 | Biosynthesis of unsaturated fatty acids | - | 0.0179 |  |
| hsa04664 | Fc epsilon RI signaling pathway | - | 0.0193 |  |
| hsa04340 | Hedgehog signaling pathway | - | 0.0194 | [59] |
| hsa04722 | Neurotrophin signaling pathway | - | 0.0216 |  |
| hsa05213 | Endometrial cancer | - | 0.0224 |  |
| hsa00531 | Glycosaminoglycan degradation | - | 0.023 |  |
| hsa04962 | Vasopressin-regulated water reabsorption | - | 0.0336 |  |
