# Supplementary material for: Predicting Disease Risk Using Bootstrap Ranking and Classification Algorithms
Source: PLoS Comput Biol. 2013 Aug 22;9(8):e1003200. doi: 10.1371/journal.pcbi.1003200 (PMC3749941; doi:10.1371/journal.pcbi.1003200)
Supplement: Table S8 — Mean test AUC-PR for different algorithms using BootRank. Shown are the average AUC values for the Precision-Recall (PR) curve for test individuals for the different algorithms when using BootRank, or when combining all 7 algorithms (Majority), or only 4 algorithms (4-Majority). The best single algorithm for each disease is highlighted in gray. (DOCX) [file pcbi.1003200.s016.docx]

| **Disease / algorithm** | **T1D** | **T2D** | **BD** | **CD** | **CAD** | **RA** | **HT** |
| --- | --- | --- | --- | --- | --- | --- | --- |
| **Support vector machine (SVM)** | **0.92** | **0.81** | **0.83** | **0.67** | **0.67** | **0.75** | **0.66** |
| **Random forest (RF)** | **0.87** | **0.80** | **0.79** | **0.77** | **0.79** | **0.79** | **0.79** |
| **Regularized logistic regression (RLR)** | **0.93** | **0.81** | **0.79** | **0.71** | **0.75** | **0.80** | **0.72** |
| **Naïve Bayes (NB)** | **0.85** | **0.87** | **0.88** | **0.74** | **0.76** | **0.78** | **0.74** |
| **Allele count (AC)** | **0.82** | **0.83** | **0.84** | **0.65** | **0.63** | **0.68** | **0.65** |
| **Log Odds (LO)** | **0.82** | **0.68** | **0.68** | **0.62** | **0.63** | **0.70** | **0.62** |
| **Robust adaboost (RAB)** | **0.91** | **0.83** | **0.81** | **0.72** | **0.79** | **0.78** | **0.75** |
|  |  |  |  |  |  |  |  |
| **Majority (all algorithms)** | **0.91** | **0.85** | **0.86** | **0.71** | **0.74** | **0.77** | **0.71** |
| **4-Majority (only RF, RLR, NB and RAB)** | **0.93** | **0.85** | **0.84** | **0.73** | **0.78** | **0.80** | **0.74** |
